# Clinical and Doppler Ultrasonographic Correlation of Varicocele in Male Infertility

**DOI:** 10.7759/cureus.95873

**Published:** 2025-11-01

**Authors:** Malvika Misra, Shubhi Srivastava

**Affiliations:** 1 Obstetrics and Gynaecology, Dr. Ram Manohar Lohia Institute of Medical Sciences, Lucknow, IND; 2 Reproductive Medicine and Surgery, Mahatma Gandhi Medical College and Hospital, Jaipur, IND

**Keywords:** color flow doppler ultrasound, male infertility, semen analysis, ultrasonography, varicocele

## Abstract

Background: Varicocele is a common, surgically correctable cause of male infertility. Its prevalence, clinical patterns, and impact on semen quality vary among populations, with limited data from India. This study aimed to assess clinical, semen, and ultrasonographic characteristics of infertile men with varicocele compared to infertile men without varicocele at a tertiary referral center in northern India.

Methods: This prospective observational study was conducted at Dr. Ram Manohar Lohia Institute of Medical Sciences (Dr. RMLIMS), Lucknow, from September 2023 to August 2024. Ninety infertile men were enrolled: 45 with clinically diagnosed varicocele (cases) and 45 without varicocele (controls). Clinical grading, scrotal Doppler parameters, and semen analyses were recorded. Group differences were analyzed using chi-square and t-tests. Odds ratios (OR) with 95% confidence intervals (CI) were calculated. Binary logistic regression was adjusted for age and type of infertility.

Results: Azoospermia was significantly more frequent in cases (46.7%) than controls (20%) (OR: 3.51; 95% CI: 1.37-9.02; p = 0.007). Oligozoospermia occurred in 60% of cases versus 35.6% of controls (OR: 2.71; 95% CI: 1.12-6.55; p = 0.020). Mean pampiniform plexus diameters were larger in cases (right: 2.15 ± 0.66 mm; left: 2.95 ± 0.72 mm) compared to controls (right: 1.30 ± 0.21 mm; left: 1.47 ± 0.19 mm; p < 0.001 for both). High-grade left varicoceles (grades IV-V) were strongly associated with azoospermia (p = 0.014) and oligozoospermia (p = 0.007). Regression analysis confirmed varicocele as an independent predictor of azoospermia (adjusted OR: 3.23; 95% CI: 1.18-8.82). Post hoc power exceeded 80% for primary outcomes.

Conclusions: In this cohort, varicocele was independently associated with impaired spermatogenesis, particularly azoospermia and oligozoospermia. Ultrasound measurements confirmed clinical findings, with larger pampiniform plexus diameters in affected men. These findings underscore the importance of routine varicocele evaluation in the workup of male infertility in India.

## Introduction

Male infertility accounts for nearly half of all couple infertility cases worldwide, with a substantial burden in low- and middle-income countries such as India. Among the potentially correctable causes, varicocele remains the most common surgically treatable factor. Varicocele is characterized by abnormal dilatation and tortuosity of the pampiniform venous plexus within the spermatic cord, often due to incompetence of the internal spermatic vein valves [1]. Its prevalence is approximately 15% in the general male population and rises to 35%-40% in men with primary infertility, reaching up to 80% in secondary infertility cases [2].

The condition impairs spermatogenesis and semen quality through multifactorial mechanisms, including scrotal hyperthermia, hypoxia, reflux of renal and adrenal metabolites, oxidative stress with increased reactive oxygen species, and disruption of the hypothalamic-pituitary-gonadal axis [3-6]. These effects lead to germ cell apoptosis, Sertoli and Leydig cell dysfunction, and deterioration in sperm concentration, motility, morphology, and DNA integrity [7,8]. Diagnosis is primarily clinical, based on scrotal examination and graded using the Dubin and Amelar system, but physical examination has limitations, especially in obese patients or those with small testes [9]. Color Doppler ultrasonography objectively assesses venous dilatation and reflux duration, detecting subclinical varicoceles and assisting preoperative evaluation [10,11]. Current guidelines from the European Association of Urology and American Urological Association recommend Doppler confirmation in suspected cases [9].

Environmental and demographic factors may influence varicocele prevalence and severity. Varicocelectomy has been shown in randomized and observational studies to improve semen parameters and pregnancy rates, especially in men with palpable varicoceles and abnormal semen [12,13]. Early detection allows timely surgical intervention while avoiding unnecessary treatment in subclinical cases. Although the contribution of varicocele to male infertility is debated due to heterogeneous studies [5,6], Indian data integrating clinical, Doppler, and semen analyses per the WHO 2021 criteria remain limited [14]. This prospective study evaluates infertile men with and without varicocele using clinical examination and Doppler ultrasound, with correlation of varicocele grade, pampiniform plexus diameter, and reflux parameters to semen quality (WHO 2021 criteria) at a tertiary care center in northern India. The aim is to provide a comprehensive profile of varicocele-associated infertility in this population.

## Materials and methods

This prospective observational study was conducted in the Department of Obstetrics and Gynaecology (Reproductive Medicine Unit) at Dr. Ram Manohar Lohia Institute of Medical Sciences (Dr. RMLIMS), Lucknow, a tertiary care referral center in northern India, from September 1, 2023, to August 31, 2024. Ninety consecutive male patients presenting to the infertility outpatient clinic during the study period were enrolled. Infertility was defined as the inability to achieve conception after at least 12 months of regular, unprotected sexual intercourse. Participants were allocated into two groups: 45 infertile men with clinically diagnosed varicocele (cases) and 45 infertile men without clinical or ultrasonographic evidence of varicocele (controls).

Eligible participants were men aged between 20 and 50 years with primary or secondary infertility who consented to undergo clinical examination, scrotal Doppler ultrasonography, and semen analysis. Men were excluded if they had a history of testicular or scrotal trauma, prior genitourinary surgery, or malignancy; current urogenital infection such as orchitis or epididymitis; known genetic causes of infertility such as Klinefelter syndrome or Y-chromosome microdeletions; a history of mumps orchitis; exposure within the preceding six months to gonadotoxic drugs (e.g., chemotherapy agents or alkylating drugs) or hormonal therapy; or systemic illnesses known to affect spermatogenesis, including uncontrolled diabetes mellitus or chronic liver disease. The study protocol was approved by the Institutional Ethics Committee of Dr. RMLIMS, Lucknow, and written informed consent was obtained from all participants prior to enrolment. Detailed history and examination were performed for all men in the presence of a male chaperone, with documentation of the duration and type of infertility.

Varicocele was diagnosed clinically by palpating the spermatic cord with the patient standing in a warm room, with and without the Valsalva maneuver. Grading was performed using the Dubin and Amelar classification: grade I (palpable only on Valsalva), grade II (palpable without Valsalva), and grade III (visible and palpable at rest).

Color Doppler ultrasonography was performed by a single experienced radiologist blinded to clinical findings, using a high-frequency (7.5-10 MHz) linear probe. Examinations were carried out in both supine and standing positions, with and without the Valsalva maneuver. The maximum pampiniform plexus vein diameter was measured three times, and the mean value was used for analysis. Reflux duration was noted, and a vein diameter of ≥2.5 mm with reflux lasting more than one second was considered diagnostic of varicocele. Testicular volume was calculated using the ellipsoid formula (length × width × height × 0.52).

Semen samples were collected after 3-5 days of abstinence by masturbation into sterile containers, allowed to liquefy at room temperature, and analyzed within one hour according to the WHO Laboratory Manual for the Examination and Processing of Human Semen, sixth edition (2021). Two semen samples were collected and analyzed for each participant. Azoospermia was defined as the absence of spermatozoa in the ejaculate, confirmed after centrifugation twice. Oligozoospermia was defined as a sperm concentration of <15 million/mL. Asthenozoospermia was diagnosed when progressive motility was <32%. Necrozoospermia indicated ≥58% immotile spermatozoa, and teratozoospermia was defined as <4% normal sperm morphology.

Data were analyzed using SPSS version 26 (IBM Corp., Armonk, NY). Continuous variables were expressed as mean ± standard deviation (SD), and categorical variables as frequencies and percentages. Normality was assessed using the Shapiro-Wilk test. Normally distributed continuous variables were compared using Student’s t-test, and Welch’s t-test was applied when variances were unequal. Categorical variables were compared using the χ² test or Fisher’s exact test as appropriate. Odds ratios (OR) with 95% confidence intervals (CI) were calculated. Binary logistic regression was used to determine whether varicocele independently predicted azoospermia and oligozoospermia after adjusting for age and infertility type. Post hoc power analysis using G*Power version 3.1 (Heinrich-Heine-Universität Düsseldorf, Düsseldorf, Germany) (two-tailed, α = 0.05) showed that the sample size provided approximately 80% power for detecting differences in azoospermia prevalence and >65% power for differences in oligozoospermia prevalence. A p-value of <0.05 was considered statistically significant.

## Results

The mean age of the participants was comparable between the two groups: 31.2 ± 5.8 years in cases and 30.9 ± 6.1 years in controls (p = 0.78). The distribution of primary versus secondary infertility did not differ significantly (p = 0.42). Duration of infertility ranged from 1 to 8 years (mean: 3.6 years). Bilateral varicocele was identified in 37.8% of cases, left-sided in 55.6%, and right-sided in 6.7%. High-grade (grade III) left varicoceles accounted for 77.8% of all left varicocele cases. Table 1 summarizes the baseline demographic and clinical findings.

**Table 1:** Baseline demographic and clinical characteristics SD: standard deviation

Variable	Cases (n = 45)	Controls (n = 45)	p-value
Mean age (years)	31.2 ± 5.8	30.9 ± 6.1	0.780
Primary infertility (%)	66.7%	73.3%	0.420
Duration of infertility (years, mean ± SD)	3.6 ± 1.8	3.4 ± 1.7	0.640
Laterality of varicocele (%)	Left: 55.6, right: 6.7, bilateral: 37.8	-	-
High-grade (grade III) left varicocele (%)	77.8%	-	-

Azoospermia was present in 46.7% of cases compared to 20% of controls, with an odds ratio of 3.51, a 95% confidence interval from 1.37 to 9.02, and a p-value of 0.007. Oligozoospermia was seen in 60% of cases compared to 35.6% of controls, with an odds ratio of 2.71, a 95% confidence interval from 1.12 to 6.55, and a p-value of 0.020. Asthenozoospermia, necrozoospermia, and teratozoospermia showed higher prevalence in cases; however, the differences were not statistically significant. Table 2 shows the comparison of semen abnormalities.

**Table 2:** Semen abnormalities in cases and controls OR: odds ratio, CI: confidence interval

Semen abnormality	Cases (number (%))	Controls (number (%))	OR (95% CI)	p-value
Azoospermia	21 (46.7)	9 (20.0)	3.51 (1.37-9.02)	0.007
Oligozoospermia	27 (60.0)	16 (35.6)	2.71 (1.12-6.55)	0.020
Asthenozoospermia	19 (42.2)	14 (31.1)	1.59 (0.68-3.72)	0.284
Necrozoospermia	7 (15.6)	4 (8.9)	1.89 (0.51-6.97)	0.336
Teratozoospermia	11 (24.4)	7 (15.6)	1.74 (0.61-4.97)	0.302

The mean right pampiniform plexus diameter was 2.15 mm with a standard deviation of 0.66 in cases, compared to 1.30 mm with a standard deviation of 0.21 in controls, with a p-value of less than 0.001. The mean left pampiniform plexus diameter was 2.95 mm with a standard deviation of 0.72 in cases, compared to 1.47 mm with a standard deviation of 0.19 in controls, with a p-value of less than 0.001. Testicular volumes did not differ significantly between groups. Table 3 summarizes the ultrasonographic findings.

**Table 3:** Ultrasonographic findings SD: standard deviation

Parameter	Cases (mean ± SD)	Controls (mean ± SD)	p-value
Right pampiniform diameter (mm)	2.15 ± 0.66	1.30 ± 0.21	<0.001
Left pampiniform diameter (mm)	2.95 ± 0.72	1.47 ± 0.19	<0.001
Right testicular volume (cm³)	12.05 ± 4.20	11.60 ± 2.90	0.556
Left testicular volume (cm³)	10.40 ± 4.00	11.20 ± 3.50	0.315

High-grade left varicocele, defined in this study as grade III on clinical examination, showed a strong and statistically significant association with severe spermatogenic impairment. Among men with high-grade left varicocele, the likelihood of azoospermia was markedly increased (57.1% versus 10.0%), with an odds ratio of 12.0, 95% CI of 1.4-100.5, and a p-value of 0.012. A similarly significant association was observed for oligozoospermia (71.4% versus 20.0%), with an odds ratio of 10.0, 95% CI of 1.7-57.8, and a p-value of 0.006.

**Table 4:** Association of high-grade left varicocele with semen abnormalities OR: odds ratio, CI: confidence interval

Abnormality	High-grade (n = 35) (number (%))	Low-grade (n = 10) (number (%))	OR (95% CI)	p-value
Azoospermia	20 (57.1)	1 (10.0)	12.0 (1.4-100.5)	0.012
Oligozoospermia	25 (71.4)	2 (20.0)	10.0 (1.7-57.8)	0.006

After adjusting for age and type of infertility, the presence of varicocele remained an independent predictor of azoospermia, with an adjusted odds ratio of 3.23 (95% CI: 1.18-8.82; p = 0.022). It was also an independent predictor of oligozoospermia, with an adjusted odds ratio of 2.58 (95% CI: 1.03-6.45; p = 0.043). Post hoc power analysis (two-tailed, α = 0.05) indicated that the study had approximately 80% power to detect the observed difference in azoospermia prevalence (46.7% in cases versus 20% in controls) and about 65%-70% power to detect the difference in oligozoospermia prevalence (60% versus 35.6%). These values reflect achieved rather than prospective power and should be interpreted with caution. Table 5 summarizes these findings.

**Table 5:** Multivariate logistic regression for semen abnormalities OR: odds ratio, CI: confidence interval

Outcome	Predictor	Adjusted OR (95% CI)	p-value
Azoospermia	Varicocele (yes versus no)	3.23 (1.18-8.82)	0.022
Oligozoospermia	Varicocele (yes versus no)	2.58 (1.03-6.45)	0.043

## Discussion

This prospective observational study from Dr. Ram Manohar Lohia Institute of Medical Sciences, Lucknow, evaluated 90 infertile men and demonstrated a significant association between varicocele and impaired semen quality. Both azoospermia and oligozoospermia were more prevalent in the varicocele group compared to infertile controls, and these associations persisted with clinically important effect sizes. Azoospermia was present in 46.7% of cases compared to 20% of controls (OR: 3.51; 95% CI: 1.37-9.02; p = 0.007), while oligozoospermia was observed in 60% of cases compared to 35.6% of controls (OR: 2.71; 95% CI: 1.12-6.55; p = 0.020).

The prevalence of azoospermia in our cohort (46.7%) is higher than most Western series, where it ranges from 15% to 30% [1,2], but is comparable to Indian data, such as those by Singh et al., who reported 42% in a similar tertiary care cohort [13]. Oligozoospermia prevalence (60%) was also within the upper range of recent international series, including a multicenter Asian cohort study by Chen et al. (54%-62%) [12]. On ultrasonography, we found significantly larger pampiniform plexus diameters in the varicocele group (mean left: 2.95 ± 0.72 mm versus 1.47 ± 0.19 mm in controls; p < 0.001), consistent with Zampieri et al., who demonstrated that a venous diameter of >2.5 mm with reflux of >1 s is an optimal diagnostic threshold [6]. These findings support Doppler ultrasonography as a valuable adjunct to clinical examination.

High-grade varicoceles (grade III) showed a strong correlation with severe spermatogenic impairment in our study. This stepwise association aligns with Elbardisi et al., who demonstrated that increasing venous reflux severity correlates with reduced sperm concentration, motility, and higher DNA fragmentation indices [7]. The biological plausibility of this relationship is supported by known mechanisms: scrotal hyperthermia and oxidative stress impair Sertoli cell function and DNA integrity [9,10], venous stasis leads to hypoxia and reactive oxygen species accumulation [11], and reflux of renal/adrenal metabolites can interfere with Leydig cell steroidogenesis [12]. Molecular studies further suggest mitochondrial dysfunction and activation of apoptotic pathways in testicular tissue from men with varicocele [13].

Clinically, these findings highlight the importance of early detection and careful grading of varicocele. The strong association with severe semen abnormalities suggests that selected patients may benefit from timely surgical intervention. Evidence from Indian cohorts, including Gupta and Kumar [14], has shown significant improvement in sperm parameters and spontaneous pregnancy rates exceeding 30% at one year after varicocelectomy. Nevertheless, surgical decision-making should remain individualized, accounting for clinical grade, semen profile, female partner’s reproductive status, and duration of infertility.

From an epidemiological standpoint, the relatively higher prevalence and severity observed in Indian men may reflect environmental exposures, delayed presentation, and underutilization of fertility evaluation services. Global reviews emphasize the need for region-specific approaches to enhance awareness, screening, and referral pathways. The present study has several strengths: a prospective design, well-defined inclusion and exclusion criteria, combined clinical and ultrasonographic assessment, and semen analysis based on WHO 2021 guidelines, which ensures comparability with international data. Effect size reporting and regression modeling enhanced the robustness of interpretation.

However, this study also has limitations and potential sources of bias. The single-center setting may limit generalizability, and convenience sampling at a tertiary care facility could introduce selection bias. The sample size, although adequate for detecting primary outcomes (post hoc power: ~80% for azoospermia and ~65%-70% for oligozoospermia), was modest. The cross-sectional design precludes causal inference, and treatment outcomes were not evaluated. Finally, unmeasured confounders such as lifestyle, occupational exposures, or genetic abnormalities may have influenced results.

## Conclusions

In this prospective observational study from a tertiary referral center in northern India, the presence of varicocele was independently associated with impaired spermatogenesis, most notably azoospermia and oligozoospermia. Ultrasonographic parameters showed that larger pampiniform plexus vein diameters and higher clinical grades of varicocele correlated with greater severity of semen abnormalities, suggesting a dose-response relationship between varicocele severity and deterioration of sperm quality. These results highlight the importance of systematic evaluation for varicocele in the workup of male infertility, using both careful physical examination and confirmatory color Doppler ultrasonography. While our findings support the potential value of early detection, particularly in high-grade cases with abnormal semen parameters, further prospective studies with treatment outcomes are needed before definitive conclusions about intervention can be drawn.
